# New Insights Into Cancer Chronotherapies

**DOI:** 10.3389/fphar.2021.741295

**Published:** 2021-12-13

**Authors:** Jingxuan Zhou, Jiechen Wang, Xiaozhao Zhang, Qingming Tang

**Affiliations:** ^1^ Department of Stomatology, Union Hospital, Tongji Medical College, Huazhong University of Science and Technology, Wuhan, China; ^2^ School of Stomatology, Tongji Medical College, Huazhong University of Science and Technology, Wuhan, China; ^3^ Hubei Province Key Laboratory of Oral and Maxillofacial Development and Regeneration, Wuhan, China; ^4^ Beijing Tongren Eye Center, Beijing Tongren Hospital, Capital Medical University, Beijing, China; ^5^ Beijing Institute of Ophthalmology, Beijing Tongren Hospital, Capital Medical University, Beijing, China

**Keywords:** circadian clocks, circadian rhythms, cancers, chronotherapies, personalized chronotherapies 3

## Abstract

Circadian clocks participate in the coordination of various metabolic and biological activities to maintain homeostasis. Disturbances in the circadian rhythm and cancers are closely related. Circadian clock genes are differentially expressed in many tumors, and accelerate the development and progression of tumors. In addition, tumor tissues exert varying biological activities compared to normal tissues due to resetting of altered rhythms. Thus, chronotherapeutics used for cancer treatment should exploit the timing of circadian rhythms to achieve higher efficacy and mild toxicity. Due to interpatient differences in circadian functions, our findings advocate an individualized precision approach to chronotherapy. Herein, we review the specific association between circadian clocks and cancers. In addition, we focus on chronotherapies in cancers and personalized biomarkers for the development of precision chronotherapy. The understanding of circadian clocks in cancer will provide a rationale for more effective clinical treatment of tumors.

## Introduction

Circadian clocks exist in biological organisms and serve as a method to adapt to natural environmental changes. Circadian clocks consist of the suprachiasmatic nucleus (SCN) and peripheral clocks. When variations in environmental light or other stimuli occur based on the laws of nature, the SCN recognizes these changes and inputs signals to specific pathways so that subordinate clocks receive the correct messages to make the corresponding adjustments to the 24-h cycle (Shafi and Knudsen, 2019; Kinouchi and Sassone-Corsi, 2020). Studies over the last decade have shown that circadian clocks regulate various physiological activities and are essential to our health. At the complex multicellular and molecular level, circadian clocks participate in the evolutionary machinery that stipulates the temporal adjustment of physiology to maintain homeostasis through the establishment of circadian rhythms. Any disruption gives rise to many chronic diseases, such as metabolic disorders and malignant tumors.

Evidence has shown there is a strong interaction between tumors and circadian clocks. The clock genes, which generate oscillatory signals transmitted to the molecular clocks, are altered in different cancers. Further, clock genes whose expression is dysregulated may act as driving factors in carcinogenesis and cancer progression. Our research group demonstrated that brain and muscle aryl hydrocarbon receptor nuclear translocator1 (BMAL expression presents rhythmic oscillation and is closely associated with the development of oral squamous cell carcinoma (OSCC). A genetically engineered mouse lung tumor model was used to demonstrate that disruption of circadian rhythms could promote lung tumor growth and decrease survival (Papagiannakopoulos et al., 2016). Oncogenic processes weaken or disrupt circadian rhythms (Huang et al., 2011). In addition, tumor tissues reset their circadian rhythms compared with normal tissues. Thus, chronotherapies could improve efficacy and alleviate biotoxicity in tumor treatments if differences in circadian rhythms are considered during drug administration.

Generally, chronotherapies depend on the circadian timing system (CTS) that controls circadian rhythms involving metabolism and biological activities (Cederroth et al., 2019). Accumulating evidence has shown that providing rhythmic treatments can not only avoid some of the side effects associated with cancer therapy but this approach can also improve prognosis, for example, administering a drug at a specific time can reduce changes in its metabolism and in patient fatigue (Ozturk et al., 2017; Shuboni-Mulligan et al., 2019; Sulli et al., 2019). However, some dosage regimens for traditional treatments are not aligned to the individual characteristics of cancer patients due to differences in circadian rhythms between normal and tumor tissues. Precise and optimal timing is required to exploit personalized chronotherapeutic delivery for each individual (Ozturk et al., 2017). Thus, potential molecular targets or biomarkers have been investigated to determine real-time dosing regimens. One such marker investigated by our group is *BMAL1*, which presents stable rhythmic oscillations and is considered a target for treatment with relevant anticancer drugs at a specific timepoint. How to identify personalized indicators that can be applied to chronotherapies has become a crucial question.

This review summarizes the expression patterns of clock genes in tumors and describes studies in which the biological activities of cancer rhythms are closely associated with circadian clocks and tumors. We also focus on the mechanisms and specific treatments using the chronotherapy approach in existing studies and applications. In addition, personalized biomarkers with constant rhythms such as *BMAL1* and temperature are of great concern. Based on these characteristics, we can provide an optimized treatment plan for individual cancer patients with improved efficacy.

## The Correlation Between the Circadian Clock and Tumor Biology

### Expression Patterns of Clock Genes are Variable in Tumors

**FIGURE 1:**
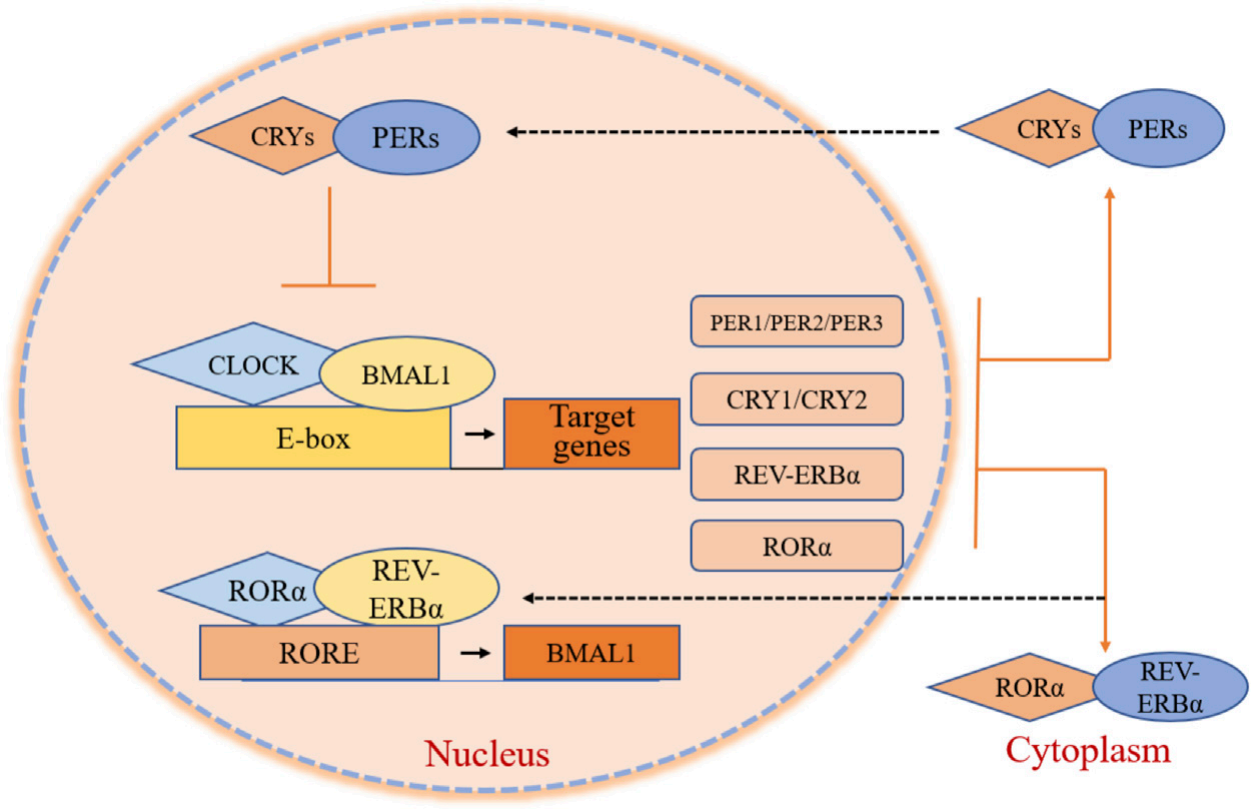
Core clock gene organization in circadian clocks. *BMAL1* and *CLOCK* are transcription factors that contain two helix-loop-helix domains and bind E-box elements in the *PER*, *CRY*, *REV-ERBα*, and *RORα* genes, influencing positively on circadian transcription. *PER* and *CRY* form heterodimers, interacting with casein kinase Iε (CKIε) and translating into the nucleus. These two genes negatively mediate *BMAL1/CLOCK*-driven transcription. *RORα* and *REV-ERBα* activate and repress the transcription of *BMAL1* through their competitive action on response elements (ROREs) on the *BMAL1* promoter.

At the molecular level, in the *BMAL1* and circadian locomotor output cycles kaput (*CLOCK*) act as transcription factors. They include two essential helix-loop-helix domains and bind E-box elements (CACGTG) in the Period (*PER*) and Cryptochrome (*CRY*) genes, which positively influence circadian transcription. *CRY* and *PER* form heterodimers that ineract with casein kinase Iε (CKIε). Both genes translocate into the nucleus to negatively mediate *BMAL1/CLOCK*-driven transcription (Figure 1) (Shearman et al., 2000). The alternations of clock gene expressions are closely connected with the occurrence and development of cancers. For brain tumors, the expression of *CLOCK* in high-grade glioma cells increases significantly compared with low-grade gliomas and non-gliomas, likely due to a decrease in miR-124 expression, which regulates RNA expression (Chen et al., 2013; Li A. et al., 2013). In one study, the expression of *PER2* and *BMLA1* significantly decreased in OSCC tumors as evaluated by reverse transcription-quantitative PCR (RT-qPCR) and immunohistochemistry (Xiong et al., 2018). The same was observed in head and neck squamous cell carcinoma (HNSCC) and in nasopharyngeal carcinoma (Hou et al., 2020; Hsu et al., 2012; Rahman et al., 2019). *CLOCK*, *PER1*, as well as *CRY* levels, are also reduced in HNSCC. Compared to normal skin, patients with skin cutaneous melanoma (SKCM) present significant down-regulation in the expression of *BMAL1*, *CRY1*, *CRY2*, *PER1*, *PER2,* and *PER3*, and higher expression of *CLOCK* (de Assis et al., 2018), a similar pattern was also observed in patients with colon adenocarcinoma (COAD) (Fuhr et al., 2018; Krugluger et al., 2007; Neilsen et al., 2019). *BMAL1* and *PER3* levels increase, and *CLOCK* and *CRY2* levels decrease in tissue samples obtained from follicular thyroid cancer (FTC) and papillary thyroid cancer (PTC) nodule tissues compared to benign tissues (Malaguarnera et al., 2020). In thymoma, only the level of *PER1* decreases, while the expression of other clock genes all increase. In endometrial endometrioid carcinoma, *PER2* is up-regulated nearly 15-fold in isolated esophageal tumors (Eca) with metastasis (Li et al., 2016). The expression of *CLOCK* is markedly increased in breast cancer, as increased expression of differentiated embryonic chondrogenic gene 1 (*DEC1)* has been associated with increased expression of estrogen receptor α (ERα), which binds to the *CLOCK* promotor to regulate its transcription (Xue et al., 2020), while *BMAL1*, *PER,* and *CLOCK* levels reduce. In previous studies, DEC1 over-expression in esophageal cancer, OSCC, and pancreatic ductal carcinoma also showed an associated increase in the expression of MIC-1, a p53-activated apoptosis factor. DEC1 interferes with cell apoptosis, and cancer cells become immortalized (Wu et al., 2012; Xu et al., 2012). However, in esophageal tumors, *CLOCK*, *PER1*, *PER2*, *PER3*, *CRY1* as well as *CRY2* levels are down-regulated and their downstream proteins accordingly become disordered (van der Watt et al., 2020). As for lung cancer cells, the primary circadian gene *CLOCK* is up-regulated in CD133+ cells, and this can also be observed in A549 and H1299 cells (Jiang et al., 2020; Yoshida et al., 2013). A comparative analysis revealed that *PER1* levels were nearly 30% those of normal tissues, *PER2* was 70%, *CRY1* was 66%, *CRY2* was 30%, and *BMAL1* was 80% in non-small cell lung cancer (Chen et al., 2020). The down-regulated expression of *PER2* is attributed to the decrease in Kmt2d expression, a Histone methyltransferase that is suppressed in lung cancer cells (Alam et al., 2020; Yu et al., 2018). The expression of *PER1*, *PER2,* and *CRY2* decrease in human fibrosarcoma and in undifferentiated pleomorphic sarcoma (Rivera-Reyes et al., 2018).The expression of *BMAL1* and *CLOCK* are reduced in ovarian cancer due to the methylation of CpG sites on gene promoter regions (Gutiérrez-Monreal et al., 2016). Further, the expression of *PER2* is reduced in ovarian cancer via inhibition of the PI3K (phosphatidylinositol 3-kinase) signaling pathway (Wang et al., 2020; Yeh et al., 2014). The altered expressions in ovarian cancer agree with adrenocortical carcinoma (ACC), cervical and endocervical cancer (CESC), uterine corpus endometrium carcinoma (UCEC), uterine carcinosarcoma (UCS), testicular germ cell tumors (TGCT), chronic myeloid leukemia (CML), liver hepatocellular carcinoma (LIHC), and prostate adenocarcinoma (PRAD) (Angelousi et al., 2020; Cao et al., 2009; Lin et al., 2008; Yang et al., 2006; Yang et al., 2009). The circadian genes show up-regulated expression in pancreatic cancer, except for *BMAL1* (Li et al., 2020; Relles et al., 2013). In contrast, the levels of common clock genes are decreased except for *PER3* and *CYR2* in cholangiocarcinoma. In kidney renal clear cell carcinoma (KIRC), the levels of *BMAL1*, *(Human) Recombinant Protein (P01) (NR1D1)*, *PER1*, and *PER2* are up-regulated, while *CLOCK* and *CRY expression* is down-regulated (Litlekalsoy et al., 2016; Zhou et al., 2020). The up-regulation of *BMAL1*, *CLOCK*, and *PER* in gastric cancer and the up-regulation of *CRY1* in more advanced stage gastric cancer but not in the earlier stage has also been reported (Hu et al., 2014). In addition, in rectum adenocarcinoma (READ), *BMAL1*, *PER1*, *PER3*, and *CRY* levels decrease while *CLOCK* and *PER2* levels increase (Lu et al., 2015). In acute myeloid leukemia (AML), *PER2*, *PER3*, and *CRY* levels are reduced due to reduced expression of CCAAT/enhancer-binding proteins (C/EBPs), while *BMAL1*, *CLOCK*, and *PER1* levels increase(Gery et al., 2005). In lymphoid neoplasms, diffuse large B-cell lymphoma (DLBC), *BMAL1*, *PER1*, and *PER2* levels are down-regulated, while *CLOCK*, *PER3*, and *CRY* levels show the opposite pattern (Table 1). Altogether, results demonstrate that the expression of clock genes varies in different types of tumor cells, and the disruption of clock rhythms is related to the occurrence and development of cancers (Gu et al., 2018; Verlande and Masri, 2019).

**TABLE 1:** Variational expression of circadian clock genes in different types of cancers.

Cancer types	Clock genes	Variation	Mechanisms	References
LGG	*BMAL1*	Down-regulated	Lack of study	GEPIA (ENSG00000133794.17)
	*CLOCK*	Up-regulated		(ENSG00000134852.14)
	*PER*	Up-regulated		(ENSG00000179094.13/ENSG00000132326.11/ENSG00000049246.14)
	*CRY1*	Up-regulated		(ENSG00000008405.11)
	*CRY2*	Down-regulated		(ENSG00000121671.11)
GBM	*BMAL1*	Up-regulated	Lack of study	GEPIA (ENSG00000133794.17)
	*CLOCK*	Up-regulated	miR-124 modulates NF-кB	(ENSG00000134852.14) (Li et al., 2013a; Chen et al., 2013)
	*PER1*	Up-regulated	Lack of study	(ENSG00000179094.13)
	*PER2*	Down-regulated	Lack of study	(ENSG00000132326.11)
	*PER3*	Down-regulated	Lack of study	(ENSG00000049246.14)
	*CRY1*	Up-regulated	Lack of study	(ENSG00000008405.11)
	*CRY2*	Down-regulated	Lack of study	(ENSG00000121671.11)
PCPG	*BMAL1*	Down-regulated	Lack of study	GEPIA (ENSG00000133794.17)
	*CLOCK*	Up-regulated		(ENSG00000134852.14)
	*PER1*	Down-regulated		(ENSG00000179094.13)
	*PER2*	Up-regulated		(ENSG00000132326.11)
	*PER3*	Up-regulated		(ENSG00000049246.14)
	*CRY1*	Down-regulated		(ENSG00000008405.11)
	*CRY2*	Up-regulated		(ENSG00000121671.11)
HNSC	*BMAL1*	Controversial	Lack of study	GEPIA (ENSG00000133794.17)
	*CLOCK*	Down-regulated	Lack of study	(ENSG00000134852.14)
	*PER*	Down-regulated	Possibly connected with PI3K/AKT pathway	(ENSG00000179094.13/ENSG00000132326.11/ENSG00000049246.14) (Hou et al., 2020)
	*CRY1*	Controversial	Lack of study	(ENSG00000008405.11)
	*CRY2*	Down-regulated	Lack of study	(ENSG00000121671.11)
OSCC	*BMAL1*	Down-regulated	cyclin β1/PI3K/AKT	Xiong et al. (2018)
	*PER*	Down-regulated	p53/MMP-2	
NPC	*PER2*	Down-regulated	ERK/p38MAPK pathway	Hou et al. (2020)
ACC	*BMAL1*	Down-regulated	Lack of study	GEPIA (ENSG00000133794.17)
	*CLOCK*	Down-regulated	Steroid hormones	(ENSG00000134852.14) (Hou et al., 2020)
	*PER*	Down-regulated	Lack of study	(ENSG00000179094.13/ENSG00000132326.11/ENSG00000049246.14)
	*CRY*	Down-regulated	Lack of study	(ENSG00000008405.11/ENSG00000121671.11)
THCA	*BMAL1*	Up-regulated	Lack of study	GEPIA (ENSG00000133794.17) (Hou et al., 2020)
	*CLOCK*	Down-regulated		(ENSG00000134852.14)
	*PER1*	Down-regulated		(ENSG00000179094.13)
	*PER2*	Down-regulated		(ENSG00000132326.11)
	*PER3*	Up-regulated		(ENSG00000049246.14)
	*CYR*	Down-regulated		(ENSG00000008405.11/ENSG00000121671.11)
THYM	*BMAL1*	Up-regulated	Lack of study	GEPIA (ENSG00000133794.17) (Hou et al., 2020)
	*CLOCK*	Up-regulated		(ENSG00000134852.14)
	*PER1*	Down-regulated		(ENSG00000179094.13)
	*PER2*	Up-regulated		(ENSG00000132326.11)
	*PER3*	Up-regulated		(ENSG00000049246.14)
	*CRY*	Up-regulated		(ENSG00000008405.11/ENSG00000121671.11)
ESCA	*BMAL1*	Down-regulated	Lack of study	GEPIA (ENSG00000133794.17)
	*CLOCK*	Up-regulated	Lack of study	(ENSG00000134852.14)
	*PER1*	Down-regulated	Lack of study	(ENSG00000179094.13)
	*PER2*	Controversial	Repress E-cadherin and enhance pHDAC1	(ENSG00000132326.11)
				Hou et al. (2020)
	*PER3*	Up-regulated	Lack of study	(ENSG00000049246.14)
	*CRY1*	Up-regulated	Lack of study	(ENSG00000008405.11)
	*CRY2*	Down-regulated	Lack of study	(ENSG00000121671.11)
BRCA	*BMAL1*	Down-regulated	ERα and Her2	GEPIA (ENSG00000133794.17)
	*CLOCK*	Controversial	DEC1 increase ERα	(ENSG00000134852.14) (Hou et al., 2020)
	*PER*	Down-regulated	Lack of study	(ENSG00000179094.13/ENSG00000132326.11/ENSG00000049246.14)
	*CRY*	Down-regulated	Lack of study	(ENSG00000008405.11/ENSG00000121671.11)
LUAC	*BMAL1*	Down-regulated	Lack of study	GEPIA (ENSG00000133794.17)
	*CLOCK*	Up-regulated	Wnt/β-catenin pathway	(ENSG00000134852.14) (Hou et al., 2020)
	*PER1*	Down-regulated	EGCG	(ENSG00000179094.13)
	*PER2*	Controversial	Kmt2d	(ENSG00000132326.11)
	*PER3*	Down-regulated	Lack of study	(ENSG00000049246.14)
	*CRY*	Down-regulated	Lack of study	(ENSG00000008405.11/ENSG00000121671.11)
LUSC	*BMAL1*	Down-regulated	Lack of study	GEPIA (ENSG00000133794.17)
	*CLOCK*	Down-regulated		(ENSG00000134852.14)
	*PER1*	Down-regulated		(ENSG00000179094.13)
	*PER2*	Up-regulated		(ENSG00000132326.11)
	*PER3*	Down-regulated		(ENSG00000049246.14)
	*CRY*	Down-regulated		(ENSG00000008405.11/ENSG00000121671.11)
SARC	*BMAL1*	Up-regulated	Lack of study	GEPIA (ENSG00000133794.17)
	*CLOCK*	Up-regulated	Lack of study	(ENSG00000134852.14)
	*PER1*	Controversial	YAP1/NF-κB	(ENSG00000179094.13) (Hou et al., 2020)
	*PER2*	Up-regulated	YAP1/NF-κB	(ENSG00000132326.11)
	*PER3*	Up-regulated	Lack of study	(ENSG00000049246.14)
	*CRY1*	Up-regulated	Lack of study	(ENSG00000008405.11)
	*CYR2*	Down-regulated	YAP1/NF-κB	(ENSG00000121671.11)
OV	*BMAL1*	Down-regulated	Lack of study	GEPIA (ENSG00000133794.17)
	*CLOCK*	Down-regulated	PI3K signaling pathway	(ENSG00000134852.14) (Hou et al., 2020)
	*PER*	Down-regulated	methylation of CpG promoters	(ENSG00000179094.13/ENSG00000132326.11/ENSG00000049246.14)
	*CRY*	Down-regulated	Lack of study	(ENSG00000008405.11/ENSG00000121671.11)
CESC	*BMAL1*	Down-regulated	Lack of study	GEPIA (ENSG00000133794.17)
	*CLOCK*	Down-regulated	*TIMELESS*	(ENSG00000134852.14) (Hou et al., 2020)
	*PER*	Down-regulated	Lack of study	(ENSG00000179094.13/ENSG00000132326.11/ENSG00000049246.14)
	*CRY*	Down-regulated	Lack of study	(ENSG00000008405.11/ENSG00000121671.11)
UCEC	*BMAL1*	Down-regulated	Lack of study	GEPIA (ENSG00000133794.17)
	*CLOCK*	Down-regulated		(ENSG00000134852.14)
	*PER*	Down-regulated		(ENSG00000179094.13/ENSG00000132326.11/ENSG00000049246.14)
	*CRY*	Down-regulated		(ENSG00000008405.11/ENSG00000121671.11)
UCS	*BMAL1*	Down-regulated	Lack of study	GEPIA (ENSG00000133794.17)
	*CLOCK*	Down-regulated		(ENSG00000134852.14)
	*PER*	Down-regulated		(ENSG00000179094.13/ENSG00000132326.11/ENSG00000049246.14)
	*CRY*	Down-regulated		(ENSG00000008405.11/ENSG00000121671.11)
LIHC	*BMAL1*	Down-regulated	Lack of study	GEPIA (ENSG00000133794.17)
	*CLOCK*	Down-regulated	Lack of study	(ENSG00000134852.14)
	*PER*	Down-regulated	methylation of CpG	(ENSG00000179094.13/ENSG00000132326.11/ENSG00000049246.14) (Hou et al., 2020)
	*CRY*	Down-regulated	Lack of study	(ENSG00000008405.11/ENSG00000121671.11)
PAAD	*BMAL1*	Down-regulated	Lack of study	GEPIA (ENSG00000133794.17)
	*CLOCK*	Up-regulated	Lack of study	(ENSG00000134852.14)
	*PER1*	Controversial	Androgen receptor	(ENSG00000179094.13) (Hou et al., 2020)
	*PER2*	Up-regulated	Androgen receptor	(ENSG00000132326.11)
	*PER3*	Up-regulated	Androgen receptor	(ENSG00000049246.14)
	*CRY*	Up-regulated	Lack of study	(ENSG00000008405.11/ENSG00000121671.11)
CHOL	*BMAL1*	Up-regulated	Lack of study	GEPIA (ENSG00000133794.17)
	*CLOCK*	Up-regulated		(ENSG00000134852.14)
	*PER1*	Up-regulated		(ENSG00000179094.13)
	*PER2*	Up-regulated		(ENSG00000132326.11)
	*PER3*	Down-regulated		(ENSG00000049246.14)
	*CRY1*	Up-regulated		(ENSG00000008405.11)
	*CRY2*	Down-regulated		(ENSG00000121671.11)
KICH	*BMAL1*	Up-regulated	*SERPINE1*	GEPIA (ENSG00000133794.17) (Hou et al., 2020)
	*CLOCK*	Down-regulated	*SERPINE1*	(ENSG00000134852.14)
	*PER1*	Down-regulated	*TIMELESS/TIPIN*	(ENSG00000179094.13)
	*PER2*	Down-regulated	Lack of study	(ENSG00000132326.11)
	*PER3*	Up-regulated	Lack of study	(ENSG00000049246.14)
	*CRY1*	Down-regulated	Lack of study	(ENSG00000008405.11)
	*CRY2*	Up-regulated	Lack of study	(ENSG00000121671.11)
KIRC	*BMAL1*	Up-regulated	Lack of study	GEPIA (ENSG00000133794.17)
	*CLOCK*	Down-regulated		(ENSG00000134852.14)
	*PER*	Up-regulated		(ENSG00000179094.13/ENSG00000132326.11/ENSG00000049246.14)
	*CRY*	Down-regulated		(ENSG00000008405.11/ENSG00000121671.11)
KIRP	*BMAL1*	Up-regulated	Lack of study	GEPIA (ENSG00000133794.17)
	*CLOCK*	Down-regulated		(ENSG00000134852.14)
	*PER1*	Down-regulated		(ENSG00000179094.13)
	*PER2*	Up-regulated		(ENSG00000132326.11)
	*PER3*	Down-regulated		(ENSG00000049246.14)
	*CRY*	Down-regulated		(ENSG00000008405.11/ENSG00000121671.11)
TGCT	*BMAL1*	Down-regulated	Lack of study	GEPIA (ENSG00000133794.17)
	*CLOCK*	Down-regulated		(ENSG00000134852.14)
	*PER*	Down-regulated		(ENSG00000179094.13/ENSG00000132326.11/ENSG00000049246.14)
	*CRY*	Down-regulated		(ENSG00000008405.11/ENSG00000121671.11)
PRAD	*BMAL1*	Down-regulated	Change in epigenome	GEPIA (ENSG00000133794.17)
	*CLOCK*	Similar	Change in epigenome	(ENSG00000134852.14) (Hou et al., 2020)
	*PER*	Down-regulated	Lack of study	(ENSG00000179094.13/ENSG00000132326.11/ENSG00000049246.14)
	*CRY*	Down-regulated	Lack of study	(ENSG00000008405.11/ENSG00000121671.11)
BLCA	*BMAL1*	Down-regulated	Lack of study	GEPIA (ENSG00000133794.17)
	*CLOCK*	Down-regulated	Lack of study	(ENSG00000134852.14)
	*PER*	Down-regulated	EGFR and p16	(ENSG00000179094.13/ENSG00000132326.11/ENSG00000049246.14) (Hou et al., 2020)
	*CRY1*	Controversial	Lack of study	(ENSG00000008405.11)
	*CRY2*	Down-regulated	Lack of study	(ENSG00000121671.11)
STAD	*BMAL1*	Down-regulated	Lack of study	GEPIA (ENSG00000133794.17) (Hou et al., 2020)
	*CLOCK*	Up-regulated		(ENSG00000134852.14)
	*PER1*	Down-regulated		(ENSG00000179094.13)
	*PER2*	Up-regulated		(ENSG00000132326.11)
	*PER3*	Down-regulated		(ENSG00000049246.14)
	*CRY1*	Up-regulated		(ENSG00000008405.11)
	*CRY2*	Down-regulated		(ENSG00000121671.11)
COAD	*BMAL1*	Down-regulated	Lack of study	GEPIA (ENSG00000133794.17)
	*CLOCK*	Up-regulated	DPD	(ENSG00000134852.14) (Hou et al., 2020)
	*PER*	Down-regulated	DPD	(ENSG00000179094.13/ENSG00000132326.11/ENSG00000049246.14)
	*CRY*	Down-regulated	Lack of study	(ENSG00000008405.11/ENSG00000121671.11)
READ	*BMAL1*	Down-regulated	Lack of study	GEPIA (ENSG00000133794.17)
	*CLOCK*	Up-regulated	Lack of study	(ENSG00000134852.14)
	*PER1*	Down-regulated	P53	(ENSG00000179094.13) (Hou et al., 2020)
	*PER2*	Up-regulated	Lack of study	(ENSG00000132326.11)
	*PER3*	Down-regulated	Lack of study	(ENSG00000049246.14)
	*CRY*	Down-regulated	Lack of study	(ENSG00000008405.11/ENSG00000121671.11)
SKCM	*BMAL1*	Down-regulated	Lack of studies	GEPIA (ENSG00000133794.17)
	*CLOCK*	Up-regulated		(ENSG00000134852.14) (Hou et al., 2020)
	*PER*	Down-regulated		(ENSG00000179094.13/ENSG00000132326.11/ENSG00000049246.14)
	*CRY*	Down-regulated		(ENSG00000008405.11/ENSG00000121671.11)
AML	*BMAL1*	Up-regulated	β-catenin	GEPIA (ENSG00000133794.17)
	*CLOCK*	Up-regulated	C/EBPs	(ENSG00000134852.14) (Hou et al., 2020)
	*PER1*	Up-regulated	Lack of study	(ENSG00000179094.13)
	*PER2*	Controversial	Lack of study	(ENSG00000132326.11)
	*PER3*	Down-regulated	Lack of study	(ENSG00000049246.14)
	*CRY*	Down-regulated	Lack of study	(ENSG00000008405.11/ENSG00000121671.11)
CML	*BMAL1*	Down-regulated	Lack of study	GEPIA (ENSG00000133794.17)
	*CLOCK*	Down-regulated	methylation of the *hPER3* promoter	(ENSG00000134852.14) (Hou et al., 2020)
	*PER*	Down-regulated	methylation of the *hPER3* promoter	(ENSG00000179094.13/ENSG00000132326.11/ENSG00000049246.14)
	*CRY*	Down-regulated	Lack of study	(ENSG00000008405.11/ENSG00000121671.11)
DLBC	*BMAL1*	Down-regulated	C/EBPs	GEPIA (ENSG00000133794.17) (Hou et al., 2020)
	*CLOCK*	Up-regulated	Lack of study	(ENSG00000134852.14)
	*PER1*	Down-regulated	Lack of study	(ENSG00000179094.13)
	*PER2*	Controversial	Lack of study	(ENSG00000132326.11)
	*PER3*	Up-regulated	Lack of study	(ENSG00000049246.14)
	*CRY*	Up-regulated	Lack of study	(ENSG00000008405.11/ENSG00000121671.11)

LGG, brain lower grade glioma; GBM, glioblastoma multiforme; PCPG, pheochromocytoma and paraganglioma; HNSC, head and neck squamous cell carcinoma; NPC, nasopharyngeal carcinoma; ACC, adenoid cystic carcinoma; ERK, extracellular signal regulated kinase; MAPK, mitogen activated protein kinase; OSCC, oral squamous cell carcinoma; THYM, thymoma; ESCA, esophageal carcinoma; BRCA, breast invasive carcinoma; Her2, human epidermal growth factor receptor 2; LUAD, lung adenocarcinoma; LUSC, lung squamous cell carcinoma; SARC, sarcoma; YAP1, Yes-associated protein 1; NF-κB, nuclear factor-κB; PI3K, Phosphatidylinositol-3-kinase; OV, ovarian cancer; CESC, cervical and endocervical cancer; UCEC, uterine corpus endometrium carcinoma; UCS, uterine carcinosarcoma; LIHC, liver hepatocellular carcinoma; PRAD, prostate adenocarcinoma; CHOL, cholangiocarcinoma; KICH, kidney chromophobe; KIRC, kidney renal clear cell carcinoma; KIRP, kidney renal papillary cell carcinoma; EGCG, Epigallocatechin-3-gallate; HCC, hepatocellular carcinoma; BLCA, bladder urothelial carcinoma; STAD, stomach adenocarcinoma EGFR, epidermal growth factor receptor; DPD, dihydropyridine dehydrogenase; TGCT, testicular germ cell tumors; COAD, colon adenocarcinoma; READ, rectum adenocarcinoma; SKCM, skin cutaneous melanoma; AML, acute myeloid leukemia; C/EBPs, CCAAT/enhancer-binding proteins; CML, chronic myeloid leukemia; DLBC, lymphoid neoplasm diffuse large B-cell lymphoma.

**FIGURE 2:**
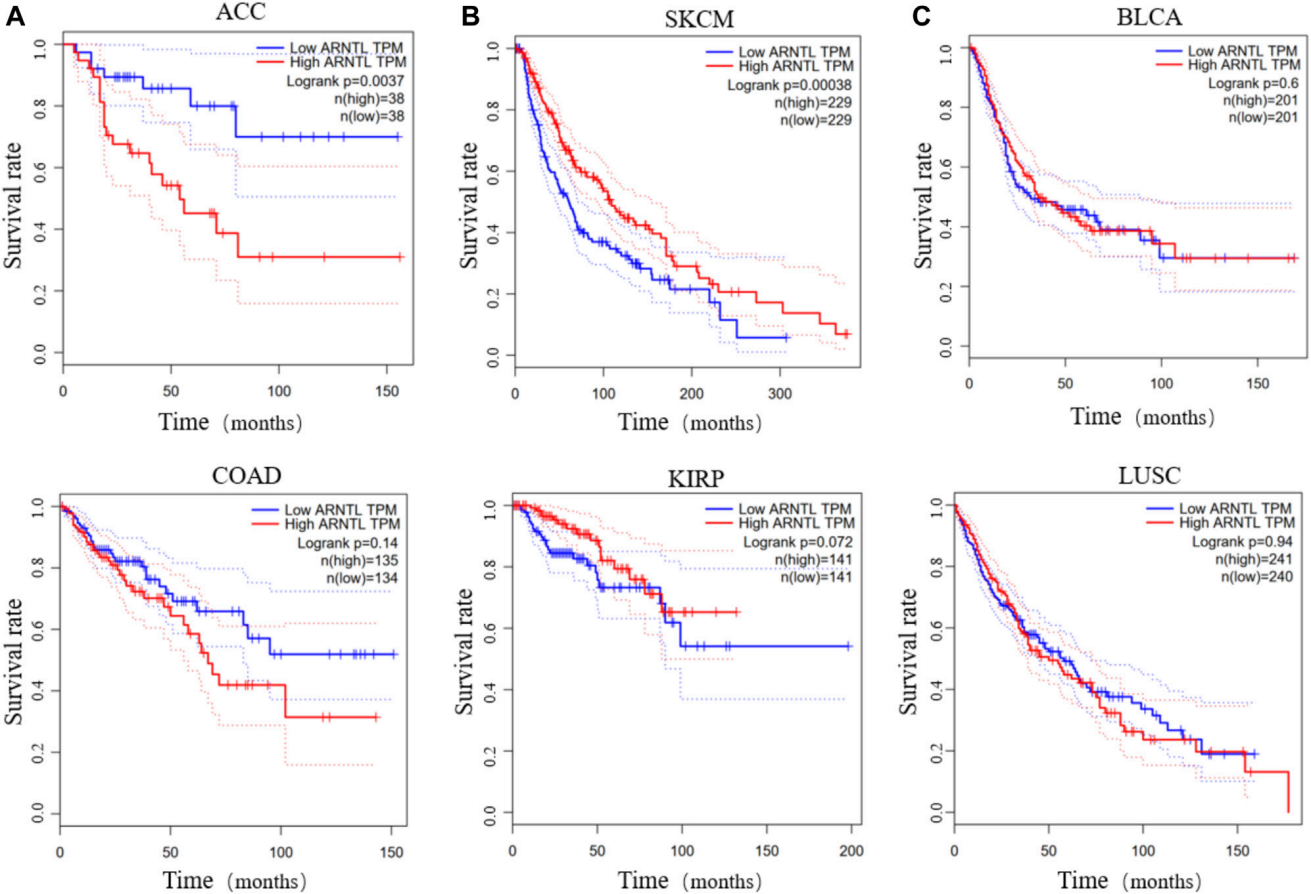
The survival rate of low and high *BMAL1* TPM in different cancers. Low *BMAL1* TPM relates to a high overall survival rate in patients with ACC or COAD **(A)**. High *BMAL1* TPM relates to a high overall survival in SKCM or KIRP **(B)**. The expression of *BMAL1* is irrelevant in BLCA or LUSC **(C)**.

In contrast, the differential expression of clock genes may be associated with prognosis and survival of cancer patients. Patients with ACC or COAD exhibit low *BMAL1* (*ARNTL*), which is associated with a higher overall survival rate over five or 10 years (Figure 2A). Inversely, patients with SKCM or KIRP show the opposite effects (Figure 2B). Furthermore, the expression of *BMAL1* is not associated with the survival of patients with BLCA and LUSC (Figure 2C). These findings suggest that the levels of clock genes play different roles across tumors and understanding their modulatory role can be advantageous to improve treatment strategies and predict the prognosis of cancer patients.

Nevertheless, there are still some issues that require further consideration, such as the oscillation in the expression of circadian clock genes in tumors. Our group tested the expression of *BMAL1* at different time points in patients with tongue squamous cell carcinoma (TSCC) after being synchronized with dexamethasone (Tang et al., 2017). The results indicated that the expression of *BMAL1* showed a stable cyclical fluctuation in TSCC. Further, *BMAL1* also exhibited a phenomenon of circadian rhythm reset, reflected by a shorter phase and reduced oscillation amplitude. In murine mammary tumor cell lines, *Per1* levels revealed a circadian rhythm with a 2.5-fold oscillation amplitude compared to normal tissues. However, in the liver, the daily maximum of *Per1* and *Per2 tumor* expression was delayed by 4 h. The oscillation amplitude of the rhythmic expression of *Cry1* decreases 5-fold in tumors, and that of *Bmal1* by 50-fold (Yang et al., 2009). These investigations suggested that clock genes may be cancer-specific and that the circadian resets of these clock genes could be closely associated with subsequent chronotherapies.

### Significant Biological activities in Tumor Tissues are Characteristic of Circadian Rhythms

As a response to the circadian reset of clock genes and the timing of circadian rhythms, the circadian clocks of tumor tissues and their cellular behaviors a differ from those of normal tissues.

The proliferation rhythms in tumor cells also differ from those of normal cells. For bone marrow cells, the highest proliferation rate peaks from the second half of the night to early in the morning, while tumor cells proliferate most actively during the first half of the night. Circadian variations in cell proliferation result from the synchronous progression of cell cycle events. The oscillation of platelet-derived growth factor (PDGF) signaling could modulate the expression of cell cycle regulators and then lead to the transition of quiescent cells into the proliferative stage (Nakagawa et al., 2008). As a result, analysis of cell-cycle distribution in malignant tissues may offer beneficial information for many chemotherapeutic agents based on phase-specificity. For example, the application of antimitotic drugs at a suitable time point can target G2/M gating of the cell cycle, which is regulated by resetting the circadian rhythm in malignant tumors. And studies show that the proliferation gene Ki-67 is regulated by the clock genes. In tumors, the circadian reset of *PER1* is related to the increase of Ki-67, accelerating cell proliferation (Ye et al., 2015). In addition, circadian clocks can regulate apoptotic processes. The altered circadian organization of B-cell lymphoma-2 (BCL-2) oncogene, can be considered a regulatory factor of programmed cell death pathways in tumors and serves as a target site for γ-radiation (Granda et al., 2005). The reset of *PERs* can inhibit the expression of BCL-2 and modulate tumor apoptosis (Li, 2019).

DNA synthesis in tumor cells results in different circadian rhythm oscillations due to the modulation by AG1295, whose mRNA accumulates nearly at the active period of DNA synthesis (Nakagawa et al., 2008). Telomerase is an important enzyme whose primary function is to maintain the correct length of the telomere. In normal organisms, there is a balance between the activity of telomerase and cell replication. Circadian clocks regulate telomerase expression, escaping damage through cellular replicative senescence to ensure cell eternity in tumor cells when circadian clocks have changed (Sulli et al., 2019). In addition, the response to DNA damage is activated in cells every day, and the level of expression of the pivotal circadian clock gene *PER1* is largely involved in DNA repair. PER1 contribute to G1/S and G2/M arrest by the Cyclin-cyclin-dependent kinase (CDK) inhibitor regulatory network (Fu et al., 2016). *CLOCK* can regulate the rhythmic profile of p53 and reset DNA damage. Thus, when the core negative regulators are down-regulated, the proper cellular response to DNA damage is disturbed (Wang et al., 2016). Further, the response of tumor cells to DNA damage differs from that of normal tissues (Sulli et al., 2019). Consequently, differences in DNA behavior due to the resetting of the circadian genes is responsible for the antimetabolic activity of drugs used in cancer chronotherapies.

When a malignancy arises, homeostatic mechanisms are dysregulated, as the energy needs of cancer cells are altered or even increased. Tumor cells reprogram their metabolism to ensure a steady supply of metabolites to generate new biomass. Therefore, these changes can be correlated with an increase in aerobic glycolysis and lipogenesis. Increased glycolysis and lipogenesis are responsible for fatty acid oxidation and methionine synthesis pathways, and both processes show circadian rhythms. In AML, UDP-glucose (Uridine diphosphate-glucose), a glycogenic precursor, is persistently upregulated. Patients with chronic lymphocytic leukemia (CLL) show high circulating levels of pyruvate and glutamate in their blood (Padmanabhan and Billaud, 2017). Based on Fuhr et al.’s research, genes involved in glycolysis and oxidative phosphorylation pathways oscillate between peak values in colorectal cancer cell lines. The oscillatory oxygen consumption rates (OCRs) show an ultradian pattern because the peak in downstream proteins present an 18-h phase shift compared to normal cells. Through unknown mechanisms synchronization occurs later, and viability and cytotoxicity decrease in the cells (Fuhr et al., 2018). KMT2D enhances *Per2* expression through super-enhancer activation and it inhibits glycolytic genes via IGFBP5-regulated insulin-like growth factor (IGF) signaling. Targeting KMT2D may contribute to rationalize glycolysis inhibition as an anticancer treatment strategy (Alam et al., 2020).

The biological behaviors of tumor cells transform to reset their circadian rhythms compared with normal cells, including cell proliferation and differentiation, DNA synthesis and replication, and metabolic activities. These processes are of great concern for the tumor process. These differences in circadian rhythms are also the biological basis of chronotherapies of cancers.

## Cancer Chronotherapies

The CTS comprises molecular clocks, driving 24-h fluctuations in detoxification and xenobiotic metabolism, immune functions, cellular proliferation, angiogenesis, apoptosis, cell cycle events, DNA repair, and many signal transduction pathways. The CTS controls drug absorption, distribution, metabolism, and excretion (ADME), which influence the chronopharmacokinetics of anticancer agents controlled by rhythmic physiology and circadian clock signaling. Conversely, the CTS also regulates cell cycle events, molecular targets, signal channels, DNA repair, and apoptosis. The CTS is responsible for the chronopharmacodynamics of anticancer drugs, radiotherapy, and other treatments with clock genes at the molecular level. The relationships between chronopharmacokinetics and chronopharmacodynamics can be used to construct optimal chronomodulated drug delivery schedules (Ozturk et al., 2017). Those cellular or pharmacologic determinants form the therapeutic potential of circadian-based chronotherapies against cancers. Essentially, chronotherapies improve the efficacy and tolerability of anticancer drugs.

### Chronopharmacokinetics of Chronotherapies

Drug pharmacokinetics govern ADME properties from the cellular to systemic levels with circadian rhythms. The ADME properties of anticancer drugs are at the mercy of large circadian variations (Levi and Schibler, 2007), such as rest-activity patterns, which could modulate blood pressure and flow to change drug absorption, cooperating with gastric pH and gastrointestinal motility (Cederroth et al., 2019). The elimination of drugs in the urine are related to several intrinsic renal variables, including glomerular filtration rate (GFR), the renal blood flow (RBF), and alkalinization/acidification degree of urine in the kidney (urine pH). Elimination mechanisms also follow the circadian rhythm of metabolic enzymes (Gachon and Firsov, 2011). Some agents that regulate gastrointestinal motility and renal variables can influence drug pharmacokinetics. For instance, brain-derived neurotrophic factor (BDNF) induces the contractility of smooth muscle cells (SMCs) by tyrosine kinase receptor B (TrkB)/RhoA/ROCK signaling. In addition, BDNF also augments noradrenergic noncholinergic (NANC) relaxation via the nitric oxide (NO)/soluble guanylate cyclase (sGC) pathway (Singh et al., 2020; Singh et al., 2021).

**FIGURE 3:**
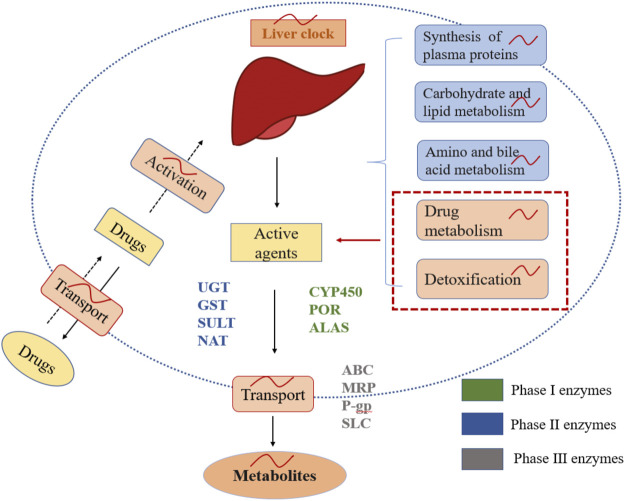
Chrono-pharmacokinetics of drug metabolism, detoxification, and delivery with metabolizing enzymes in the liver. The liver is controlled by circadian clocks that account for chrono-pharmacokinetics. Drug metabolism, detoxification, and delivery with metabolizing enzyme families are rhythmic in the liver.

Circadian rhythms of drug metabolism, detoxification, and drug transport are closely associated with drug-metabolizing enzyme families (Figure 3). Phase I and II drug-metabolizing enzyme families contain cytochrome P-450 (CYP450), aminolaevulinic acid synthase (ALAS), P450 oxidoreductase (POR), UDP-glucuronosyl transferase (UGT), glutathione-S-transferase (GST), N-acetyl transferase (NAT), and sulfotransferase (SULT). They play critical roles in oxidation, reduction, hydrolysis, and conjugation reactions (Gachon and Firsov, 2011). CYP450 catalyzes oxidative biotransformation in numerous drugs, and account for approximately ∼75% of their overall metabolism (Zanger and Schwab, 2013). All these enzymes are regulated by clock genes such as *CLOCK* and *BMAL1,* and by the circadian clock-regulated PAR-domain basic leucine zipper (RARbZip) transcription factor family (Gachon et al., 2006; Kang et al., 2007; Zhang et al., 2009; Tanimura et al., 2011). In addition, these enzyme families participate in hepatic metabolism, the liver being the primary organ of drug metabolism and detoxification and is characterized by its capacity of producing its circadian rhythms (Turek and Allada, 2002). Studies have also shown that the circadian clocks control *Cyp2a5*, *Cyp2b10*, and *Cyp3a11* mRNA levels in the liver or intestinal tract of mice. Cyp4a isoforms in the liver modulate renal function by catalyzing the formation of 20-hydroxyeicosatetraenoic acid, efficiently influencing tubular ion transport and renal vasculature, which might explain the circadian rhythms of blood pressure and renal function (Ohdo et al., 2019). The glutathione content in the liver is possibly higher in the second half of the dark span of the light-dark cycle. Suppressing the synthesis of glutathione could alter the chrono-tolerance patterns of oxaliplatin and cisplatin. In contract, UGT contents are higher during the dark span in mice. It relates to the chrono-tolerance of seliciclib and irinotecan. Phase III metabolizing enzyme families consist of ATP-Binding Cassette (ABC) transporters, including P-glycoprotein (P-gp), multi-drug resistance-associated proteins (MRP), and solute carrier (SLC) superfamily. These enzymes are responsible for transporting drugs in to and out of target tissues and cells (Gachon and Firsov, 2011). The expressions of ABC and SLC transporters are rhythmic in the liver and the intestine of mice. The ABCB1a and ABCB1b mRNA rhythms further increase the activity of P-gp during the dark span in mice. In short, changes in circadian rhythms in multifold enzymes and transporter function can potentially lead to 24-h changes in ADME properties of anticancer drugs and then lead to differences in daily bioavailability of drugs between organisms. Furthermore, changing or identifying the optimal time for the expression of specific enzymes or transporters may improve the tolerability and curative effects of anticancer drugs.

### Chrono-Pharmacodynamics of Chronotherapies

Drug activities are mainly under the control of the CTS at the molecular level, with several molecular drug targets involved in several biological functions, including cell cycle events, DNA repair, angiogenesis, intracellular targets, and triggered pathways (Singh et al., 2016; Ballesta et al., 2017). Circadian clocks mediate cell cycle events that cooperate with clock genes and signaling pathways in oncogenesis, either transcriptionally or via protein–protein interactions. Studies show that *BMAL1* inhibit cyclin E transcription to block the formation of the Cyclin E/CDK (cyclin-dependent kinase) complex, leading to cell cycle arrest in G1/S. Wee1 inactivates the CDK1/CycB complex by phosphorylation to eliminate inhibition of pCDK1 to improve G2/M phase transition (Soták et al., 2014). Dallmann et al. also demonstrated that DNA synthesis in the S phase and mitosis rhythms occur in hematopoietic cells, immune system cells, and gastrointestinal tract cells. This process targets cancer-related signal transduction pathways such as epidermal growth factor receptor (EGFR) and Rat sarcoma/rapidly accelerated fibrosarcoma/mitogen-activated protein kinase (Ras/Raf/MAPK). Furthermore, drugs targeting tubulin and microtubule in the G2/M phase are also implicated in different chronotherapy approaches. Kumar et al. showed that modulation of tubulin-microtubule dynamics interacts with vascular endothelial growth factor (VEGF) and hypoxia inducible factor-α (HIF-α) to reduce mice mammary carcinoma volumes by 48.2% (Srivastava et al., 2020). In addition, the clock genes could also be involved in apoptosis by modulating signaling pathways. *BMAL1* can suppress the expression of p21 indirectly via the retinoic acid-associated orphan receptor α (RORα) pathway (Kelleher et al., 2014). Methionine aminopeptidase 2 (MetAP2) positively regulates endothelial cell proliferation during tumor angiogenesis. *The CLOCK/BMAL1* heterodimer in the clock feedback loop enhances the transcription of the MetAP2 promoter, while *PER2*/*CRY1* inhibits promoter activation (Nakagawa et al., 2004). Furthermore, clock genes can regulate DNA repair and damage. Increasing DNA damage and genome instability has been observed in *Cry2*-deleted cells, suggesting a specific role for *Cry* in the transcriptional regulation of DNA repair. Furthermore, CRY activates the ATM and Rad3-related/creatine phosphokinase1 (ATR/CHK1) signaling pathways of DNA damage with Timeless protein (TIM), and PER1 also plays a significant role in the Ataxia telangiectasia mutated creatine phosphokinase2 (ATM-CHK2) protein complex to activate the DNA double-strand break process. All of the above biological functions can be considered molecular targets of anticancer drugs able to suppress the cancer process. Due to the regulation of circadian rhythms, it is possible to reduce side effects and improve efficacy by administering drug treatments that are optimally timed and using the proper doses. In short, chronopharmacokinetic and chronopharmacodynamics constitute an area of chronopharmacology providing a mechanistic rationale to successful treatment outcomes.

### Chrono-Chemotherapy, Targeted Therapy, and Radiotherapy Applications

Chemotherapy is one of treatment approach for tumors. To date, studies have observed the correlation between chronotherapies and chemotherapy and have identified relevant chemotherapy drugs for chronochemotherapy. Okazaki et al. used tumor-bearing mice as a model and determined that the survival rate of mice was higher when treatment was administered everolimus at 7:00 p.m. rather than at 7:00 a.m. (Okazaki et al., 2014). Another study showed that the antitumor effect of interferon (IFN)-β in nocturnally active mice was more efficient during the early rest phase than during the early activation phase (Takane et al., 2000). Imatinib is an antitumor agent that suppresses the activity of multifarious receptors with tyrosine kinase activity, also known as a protein receptor tyrosine kinase inhibitor (PKTI). Imatinib mesylate (50 mg/kg) administered during the early light phase reduces tumor growth (Nakagawa et al., 2006). Furthermore, many other anticancer drugs in which administration in line with the timing of the circadian improves antitumor efficacy have been reported, including antimetabolites (e.g., 5-fluorouracil, l-alanosine, gemcitabine, floxuridine) (Li et al., 2006; Li et al., 2005; von Roemeling and Hrushesky, 1990; Wood et al., 2006); topoisomerase Ⅰ inhibitors (e.g., irinotecan, topotecan, and 9-aminocamptothecin) (Kirichenko and Rich, 1999; Granda et al., 2002; Mullins et al., 2005); topoisomerase Ⅱ inhibitors (etoposide) (Lévi et al., 1985); alkylators (e.g., oxaliplatin, cyclophosphamide, melphalan, carboplatin, and nedaplatin) (Halberg et al., 1980; Scheving et al., 1980; Boughattas et al., 1990; Granda et al., 2002; Cui et al., 2004); cytokines (e.g., INF-α, INF-γ, INF-β, interleukin-2, BDNF) (Kemeny et al., 1992; Takane et al., 2000; Shinohara et al., 2008; Singh et al., 2021); hormones (e.g.tamoxifen) (Binkhorst et al., 2015), mitotic inhibitors e.g., docetaxel and vinorelbine (Filipski et al., 1999; Granda et al., 2001), DNA intercalators (e.g. doxorubicin) (To et al., 2003), cell cycle inhibitors (e.g. seliciclib, paclitaxel, diosgenin derivatives, analogues of 2-methoxyestradiol (2ME2), neolignans 10, and curcumin mimic 6a) (Iurisci et al., 2006; Sathish Kumar et al., 2014a; Sathish Kumar et al., 2014b; Hamid et al., 2014; Tang et al., 2017; Khwaja et al., 2018); cyclooxygenase or COX-2 inhibitors (e.g., celecoxib) (Blumenthal et al., 2001); and VEGF inhibitor (e.g., TNP-470 or O-(chloroacetyl-carbamoyl) fumagillol)), the matrix metalloproteinase inhibitor BB2516), and the VEGFR-2 TKI SU1498 (Koyanagi et al., 2003a; Koyanagi et al., 2003b). Generally speaking, cancer chronotherapies consider the association of anticancer drugs with treatment in line with a patient’s circadian rhythm. Therefore, studying the rhythmicity of these drugs provides enhanced treatments for cancers.

Targeted therapy of tumors aims at recognizing specific antigens on the surface of tumor cells through monoclonal antibodies. Conversely, it also aims at suppressing tumor cell growth through small molecules that block intracellular signal transduction by tyrosine kinases. These intracellular signals include extracellular signal-regulated kinase/mitogen-activated protein kinase (ERK/MAPK), Janus kinase (JAK), PI3K, estrogen receptor (ER), and EGFR (Firer and Gellerman, 2012). Targeted therapy has also observed circadian rhythms. Lapatinib proves to be a clock-targeted drug inhibitor of EGFR in breast cancer, acting on everolimus. Everolimus is an inhibitor of the Mammalian target of rapamycin (mTOR) through the EGFR/Ras/Ras/MAPK pathway (Pagani et al., 2011; Azzi et al., 2014; Zappe et al., 2015). Besides, lapatinib also improves the survival rate of cancer patients when administered in patients overexpressing mTOR (Okazaki et al., 2014). Other chronotarget drugs, such as erlotinib, as the EGFR inhibitor for lung cancer, have also already been reported. Erlotinib displays anti-tumor activities more effectively on tumor growth inhibition when administered in the early-light than in the early-dark phase of the circadian rhythm when EGFR activities and its downstream factors increase (Li XM. et al., 2013; Lin et al., 2015). Benzylidene indanone 1 induces apoptosis in DU145 cells by cleavage of poly ADP-ribose polymerase (PARP) (Singh et al., 2015). Hamid et al. determined that diosgenin and related compounds induce apoptosis in DU145 prostate cancer cells through the caspase pathway (Hamid et al., 2017). Targeted therapy for specific cancer sites is based on selective and available properties of cancers and normal cells with the context of the circadian clocks.

Similarly, radiation therapy is also associated with circadian rhythms influencing the expression of clock genes such as *BMAL1*, *CLOCK*, *PER*, and *CRY*. The clock genes coordinate molecular events and generate circadian rhythms influencing radiotherapy throughout the 24-h period. It predisposes cells to be more sensitive to treatments at a specific period (Palombo et al., 2015). Chronological exposure to radiation and DNA damaging agents, such as temozolomide, has an significant impact on the survival of brain tumor cells in culture (Stupp et al., 2005). Other studies also show that chrono-modulated radiation, either alone or with other drugs, can be responsible for improving therapeutic efficacy (Akgun et al., 2014). However, chrono-radiotherapy may also cause various side effects, such as long-lasting sleep disruption, cognitive defects, hair loss, and dermatitis. Therefore, the current attention to chrono-modulated administration of radiation has addressed how to reduce treatment-related symptoms. A study published by Noh et al. treated 395 patients with radiotherapy in the early morning and early evening and found that the early evening-treated group experienced significantly higher acute skin reactions compared to the morning treated group (Noh et al., 2014). Other studies demonstrated that exposure to radiation, resulted in greater hair loss in the morning and that Xpa levels were lower compared to those in the evening. These results indicate that studying the side effects of radiation chronotherapy to improve the therapeutic efficacies in tumor cells is warranted.

An increasing number of treatments support the therapeutic utility of circadian rhythms in cancer treatment This approach focuses on optimizing drug tolerability and efficacy and improving quality of life in cancer patients (Table 2).

**TABLE 2:** The correlation between anticancer drugs and circadian rhythmicity from studies.

Classification	Drugs	Cancer types	Schedule	Evidence	References
Antimetabolite	5-fluorouracil	Colon cancers	at 4:00 a.m.	Meta-analysis	Wood et al. (2006)
	L-alanosine	Lymphocytic leukemia	the half of the activity span	Animal study	Li et al. (2006)
	Gemcitabine	Osteosarcoma	3-day interval	Animal study	Li et al. (2005)
	Floxuridine	Breast cancer	the late activity-early rest span	Animal study	von Roemeling and Hrushesky, (1990)
	Capecitabine	Rectal cancer	at 8:00 a.m.&12:00p.m.	Phase II study	Akgun et al. (2014)
Top Ⅰ inhibitor	Irinotecan	Colorectal cancer	at 5:00 a.m.	PK study	Granda et al. (2002)
	Topotecan	Breast cancer	at 3:00 a.m.	Animal study	Mullins et al. (2005)
	9-AC	Breast cancer	the rest phase	Animal study	Kirichenko and Rich, (1999)
mTOR inhibitor	Everolimus	Renal cell carcinoma	at 19:00 p.m.	Animal study	Okazaki et al. (2014)
PKTI	Imatinib	Sarcoma/Melanoma	the early light phase	Animal study	Nakagawa et al. (2006)
	Lapatinib	Sarcoma/Melanoma	high expression of mTOR	Animal study	
Cytokines	Interferon α	Melanoma	at 12:00 a.m. to 04:00 a.m.	Animal study	Takane et al. (2000)
	Interferon γ	Melanoma	at 16:00 p.m.	Animal study	Takane et al. (2000)
	Interleukin-2	Hepatoma	day cycle	Animal study	Kemeny et al. (1992)
Alkylator	Cisplatin	NSCLS	at 6:00 a.m. & 18:00 p.m.	Randomized controlled study	Boughattas et al. (1990)
	Oxaliplatin	Colon cancer	at 16:00 p.m.	Animal study	Granda et al. (2002)
	CTX	Leukemia	at 11:00 a.m.	Animal study	Scheving et al. (1980)
	Carboplatin	NSCLS	at 20:00 p.m.	Toxicity analysis	Boughattas et al. (1990)
Hormones	Tamoxifen	Breast cancer	at 8:00 a.m. /13:00 p.m. /20:00 p.m.	Animal/clinical study	Binkhorst et al. (2015)
DNA intercalator	Doxorubicin	Adenocarcinoma	middle of the rest span	Animal study	To et al. (2003)
Mitotic inhibitor	Docetaxel	Adenocarcinoma	middle of the rest span	Animal study	Granda et al. (2001)
	Vinorelbine	Leukemia		Animal study	Filipski et al. (1999)
Cell cycle inhibitor	Seliciclib	Osteosarcoma	the early light phase	Animal study	Iurisci et al. (2006)
	Paclitaxel	TSCC	BMAL1 overexpression	Cell study	Tang et al. (2017)
Cox-2 inhibitor	Celecoxib	Breast cancer	at 5:00 a.m. to 1:00 p.m.	Animal study	Blumenthal et al. (2001)
VEGF inhibitor	SU1498	Lung carcinoma	early of the rest span	Animal study	Koyanagi et al. (2003a)
	BB2516	Lung carcinoma	early of the rest span	Animal study	
Radiation	γ-radiation	Rectal cancer	at 8:00 a.m.&12:00p.m.	Phase II study	Akgun et al. (2014)

9-AC, 9-aminocamptothecin; NSCLC, non-small cell lung cancer; CTX, cyclophosphamide; TSCC, tongue squamous cell carcinoma; Cox-2, cyclooxygenase-2; BB2516, matrix metalloproteinase inhibitor; SU1498, VEGFR-2, tyrosine kinase inhibitor.

### Combination of Clinical Chronotherapy Schedules

In particular, chronotherapy aims to maximize antitumor effects and to minimize the toxicity of anticancer agents in normal tissues. Patients who received chrono-modulated infusions of 5-FU, leucovorin, and oxaliplatin at separate times showed a low frequency of side effects (Lévi et al., 1992; von Roemeling and Hrushesky, 1989). In their study, Levi et al. compared the efficacy of chrono-modulated infusion to the standard fixed infusion. Patients received the 5-FU chrono-modulated infusion with administration maximum at 4:00 a.m. and the oxaliplatin administration time was maximum at 16:00. This three-drug chronomodulated regimen (chrono-FFL) produced a 58% response rate in 93 patients with metastatic colorectal cancer (Lévi et al., 1995). Taking Capecitabine 50% dose at 8:00 a.m. and 50% dose at midnight with radiotherapy decreases the toxicity of hand-foot syndrome, thrombocytopenia, diarrhea, and mucositis. Subsequently, the median survival rate was also prolonged (Akgun et al., 2014). Cisplatin often combinates with doxorubicin. Patients with ovarian or bladder cancer in a phase-II trial confirmed the better tolerability of morning doxorubicin and evening cisplatin (Innominato et al., 2010). The improvements of chrono-tolerability reduce toxicity or side-effects with conventional schedules. Chronomodulated delivery, therefore, enables the therapeutic efficacy to increase.

## Personalized Biomarkers for Precision Chronotherapies

### Temperature, Rest-Activity, and Some Hormones are Biomarkers

The SCN, a main circadian oscillator of the CTS, produces behavioral rhythms and synchronizes clocks in peripheral organs, causing measurable and therapeutically available circadian oscillations. Therefore, the rhythms of body temperature, hormone secretion, autonomic nervous system activity, cytokine release, and resting-activity form a dynamic physiological network that interacts with peripheral clocks. These rhythms serve as biomarkers of the CTS to optimize precise chronotherapy timing and dosing in individual cancer patients, respectively. Temperature forms a biomarker for cancer chemotherapy (Lévi et al., 2010). The monitoring of skin surface temperature could provide supplementary information regarding circadian phase and the CTS robustness. Patients maintain robust circadian rhythms with consistent 24-h temperature amplitudes and resting-activity after chronotherapy due to drug delivery patterns that involve administration at proper times and anti-cancer drug dose. However, chronotherapy may induce a transient or continuous circadian disturbance in the other patients, may due to the poor timing of the single or fixed chronotherapy protocols during the internal phases of those patients, which can reduce the antitumor efficacy and tolerability of anticancer therapies (Roche et al., 2014). In other clinical trials, cervical and lung cancer patients were randomly divided into several groups and received the same radiotherapy but with varying body temperatures. The growth of tumor cells was inhibited when radiotherapy was applied at the peak of body temperature. In general, resting-activity, body temperature, saliva cortisol levels, plasma catecholamines, and melatonin levels, among the various circadian biomarkers, show statistically significant and consistent rhythms in patients with early or advanced colon, lung, ovary, breast, prostate, or head and neck cancers (Lévi et al., 2010). Measurement of these biomarker patterns could help to deliver CTS information on robustness, synchronization, and internal clock timing. Confirming the peak or trough in these patterns can identify the optimal internal timing for chronotherapy delivery in individual patients.

### *BMAL1* and *PER* Serve as Biomarkers

At the molecular level, some transcription-translation feedback loops with self-regulating functions also maintain the stability of circadian rhythms. The central feedback loop consists mainly of clock genes, such as *BMAL1, CLOCK*, and downstream genes, including *PER* and *CRY* (Panda et al., 2002). Clock genes affect physiological functions of the whole body in tissues and cells specifically through multiple signal pathways, which account for interindividual differences in relevant circadian rhythms. *BMAL1* is an important transcription factor in this feedback loop that may suppress the growth, proliferation, and proliferation of multiple cancer cells, including tongue squamous cancer cells, colorectal cancer cells, breast cancer cells, and ovarian cancer cells (Gréchez-Cassiau et al., 2008; Hanahan and Weinberg, 2011; Elshazley et al., 2012; Hsu et al., 2012). Furthermore, *BMAL1* expression influences the sensitivity of chemotherapeutic drugs, including irinotecan (Dulong et al., 2015), oxaliplatin (Zeng et al., 2014), PTX (Tang et al., 2017), and cyclophosphamide. According to studies, *BMAL1* acts directly on DNA telomeres to recruit EZH2 for combining with TERT promoter to negatively regulate TERT transcription and increase TERT expression, which leads to the improvement of PTX sensitivity of TSCC. Furthermore, the efficacy of PTX in TSCC is highly consistent with the expression of *BMAL1*. The higher expression of *BMAL1*, the better the efficacy of PTX. In addition, since *BMAL1* expression exhibits a stable circadian rhythm in healthy individuals and in cancer cells, *BMAL1* could serve as a direct molecular target for PTX to determine the best drug administration time for personalized chronotherapies (Tang et al., 2017) and may contribute to improving the efficacy of anticancer drugs and reduce their toxicity.

Besides *BMAL1*, period genes (*PER1* and *PER2*) as clock genes having expression synchronized with steady circadian rhythms also influence the progression of tumors. The expression of these genes decrease in patients with pancreatic carcinoma (Relles et al., 2013), head and neck neoplasm (Hsu et al., 2012), or breast cancer (Winter et al., 2007). Niu et al. used an animal model of brain glioma to study the expressions of *Per1* and *Per2* in normal and glioma tissues, and determined that the expression of *Per1* was at its minimum at midnight, while the expression of *Per2* is maximal at midnight and minimum at 8:00 a.m. in glioma tissues (Zhanfeng et al., 2015). Another study treated animals with radiotherapy at different time points based on the disparate expression of *Per1* or *Per2* (Zhanfeng et al., 2015). The results showed that the apoptosis rate of glioma cells was higher when radiotherapy was administered at the peak of *Per1* or *Per2* expression, indicating that clock genes could act as molecular targets that regulate chronotherapy efficacy and promote personalized therapeutic effects.

Precise biomarkers or targets with constant rhythms in individuals can help identify personalized circadian rhythms in order to optimize treatment plans and thus, maximize treatment efficacy and improve quality of life.

## Conclusion and Future Prospects

Biological clocks are closely associated with the occurrence and development of tumors. Chronotherapies that exploit circadian rhythms in experimental and preclinical trials are based on the development of therapeutic interventions that consider the influence of treatment mechanisms of action and the circadian status of the tumor targets. More importantly, personalized precision chronotherapies combined with biomarkers can optimize the effective management of the administration of the chronotherapy, in clinical trials and ultimately in individual care in cancer patients. The personalized precision chronotherapy approach is characterized by the maximum therapeutic effect achievable, minimum side effects, and relatively higher potential risk prediction. Further studies should identify other biomarkers able to ameliorate personalized chronotherapies, and to provide precise chronotherapy strategies. In addition, a neoteric approach should be developed to exploit drugs that target the circadian clocks to improve efficacy and prognosis.
